# Functional soft tissue regeneration involving extremity tendons using ovine forestomach matrix grafts

**DOI:** 10.1093/jscr/rjaf1119

**Published:** 2026-01-27

**Authors:** Sandeep Naphade, Brandon A Bosque, Adam Young

**Affiliations:** Department of Plastic and Reconstructive Surgery, Pushp Superspeciality Clinic, Pune, India; Department of Medical Affairs, Aroa Biosurgery Limited, Auckland, New Zealand; Department of Medical Affairs, Aroa Biosurgery Limited, Auckland, New Zealand

**Keywords:** OFM, ovine forestomach matrix, exposed tendon, soft tissue reconstruction, xenograft

## Abstract

Soft tissue defects with exposed tendons present unique challenges in surgical reconstruction, as tendons are vulnerable to desiccation, infection, and necrosis. We present a single-center, retrospective case series of 10 patients with exposed tendons who underwent surgical reconstruction with ovine forestomach matrix (OFM) grafts. Medical records were reviewed to establish patient demographics, co-morbidities, injury characteristics, and post-operative outcomes. OFM grafts were applied for staged reconstruction, for closure via secondary intention, as implants with primary closure or fasciocutaneous flaps, and as a circumferential tendon-protective layer. The median time to granulation tissue coverage was 3 weeks (IQR: 2, 4), and to full epithelialization 4 weeks (IQR: 1, 6). The median follow-up period was 29 weeks (IQR: 6, 61). Long-term assessments evaluated tissue pliability and active range of motion. Overall, OFM provided good surgical utility, promoting rapid coverage, wound closure, and favorable functional outcomes in complex tendon exposure cases.

## Introduction

Exposed tendons within soft tissue defects are notoriously difficult to manage given their susceptibility to desiccation, infection, and necrosis. The primary goal of reconstruction is to provide rapid tissue coverage to preserve tendon function [1]. Coverage is crucial, since tendons are relatively superficial, fibrous structures, with limited blood supply, making them susceptible to dehydration and poor gliding [1].

Following debridement, infection control, and moisture balance efforts, reconstructive approaches to provide prompt tissue coverage over exposed tendons include flaps, primary closure, staged closure for split-thickness skin grafting (STSG) and the use of dermal matrices [1]. Notably, flap-based reconstruction provides immediate coverage, but may require advanced microsurgery, donor sites, and carries risks of scarring, adhesion formation, and functional loss [1–4]. Also, closure by primary intention alone may lead to the creation of dead space, increasing the risk of hematoma, seroma, and infection [5, 6]. Furthermore, staged reconstruction requires a bed of granulation tissue to support efficient inosculation of the STSG.

To address these challenges, dermal matrices may be deployed to aid soft tissue regeneration over exposed tendons. Since fibrosis can restrict tendon motion, materials that elicit a pro-regenerative immune response, such as extracellular matrix (ECM) grafts, are preferred over synthetic options [7]. Ovine forestomach matrix (OFM) is a decellularized ECM graft derived from sheep that has been used extensively in soft tissue repair, including successful coverage of exposed bone and tendons, with limited complications [8–11]. Its preserved native structure supports cell adhesion, migration, differentiation, and vascularized tissue formation that facilitates primary closure, flap advancement, and skin grafting [12–16]. OFM’s versatility for both topical and implant applications makes it suitable in various reconstructive procedures to achieve tendon coverage. This study reports our initial experience with OFM for tendon coverage, focusing on functional recovery.

## Methods and materials

### General

All patients, or their legal guardians, provided prior written informed consent for the use of their de-identified data for educational purposes. The study was conducted in accordance with World Medical Association Declaration of Helsinki ethical guidelines. Descriptive statistics (mean, median, and standard error) were computed using GraphPad Prism (v9.0.0, GraphPad Prism LLC). The Shapiro-Wilk test was applied to assess the normality of the data set with a p-value greater than 0.05 indicating that the data did not deviate significantly from a normal distribution.

### Data collection

Patient records were retrospectively reviewed from November 2022 to January 2025 to identify patients who had presented at a single institution for reconstruction of a soft tissue defect that involved exposed tendon. Only patients who had received an OFM graft (Myriad Soft Tissue Matrix™, Aroa Biosurgery Limited, Auckland, New Zealand) as part of the reconstruction were considered in the analysis. A chart review was conducted to establish patient demographics, co-morbidities, and the nature of the soft tissue injury, intra-operative notes, as well as post-operative outcomes (e.g. time to granulation tissue coverage, % STSG take at 1 week, time to healing, complications).

### Surgical procedure

All surgeries were conducted by a single surgeon (S.N.). If active infection was suspected, patients were treated with oral and/or topical antibiotics per institutional protocols. All defects were adequately debrided prior to application of OFM grafts. The grafts were used according to the instructions for use. In two defects, the OFM graft was cut to size, rehydrated then placed around the exposed tendon to create a circumferential tendon-protective layer. Alternatively, the grafts were placed on top of the exposed tendon. Grafts applied as a dermal template were secured to the peri-wound area with 2-0 polyglactin 910 sutures (Vicryl®, Ethicon, Inc., Raritan, NJ, USA). Due to the concern of infection, inflammation, and scarring associated with implanted polyglactin 910 suture [17], OFM grafts that were applied in the defect as an implant were secured to the subcutaneous tissue utilizing 2-0 poliglecaprone 25 sutures (Monocryl®, Ethicon, Inc, Raritan, NJ, USA) prior to skin closure over the OFM grafts. Defects were closed either via a fasciocutaneous flap, primary intention, secondary intention, or staged to receive an STSG (Table 2). Defects closed via secondary intention or staged were dressed with a non-adherent layer, then either a foam dressing, or a negative pressure wound therapy (NPWT) interface dressing. NPWT was conducted at 125 mmHg, per institutional protocols, and all dressings were changed weekly. Staged defects received an STSG when adequate granulation tissue coverage had formed over the tendon.

## Results

Retrospective data were collected from the medical records of 10 eligible participants. Mean patient age was 40 ± 22 years (range, 7–70 years) (Table 1). The soft tissue defects included traumatic wounds (50%), soft tissue infections (40%), and a single tumor resection (10%). The median age of the defects was 1 (interquartile range, IQR: 0, 6) week at the time of reconstruction (Table 1). The anatomic locations of defects included the lower leg and knee (40%), foot (30%), hand (20%), and arm (10%). Half of the participants had failed a previous surgical intervention (Table 1). Five (50%) participants underwent a staged reconstruction with the application of an STSG once tissue coverage of the exposed tendon had been achieved (Table 2). Three participants were reconstructed with a primary closure, where the OFM graft was implanted under the primary incision. In two of these instances, the OFM graft was placed circumferentially around the tendon(s) (Table 2). One patient was allowed to close via secondary intention once tissue coverage had been attained, and another patient was closed via a fasciocutaneous tissue flap. Two defects received post-operative NPWT for 2–3 weeks, and the median number of product applications across the cohort was 1 (IQR: 1, 1).

**Table 1:** Patient demographics and defect etiology.

Participant ID	Age	Gender	Co-morbidities/complicating factors	Defect etiology	Defect location	Prior surgical interventions	Tendon involvement	Approximate defect age (weeks)
1	21	Male	None	Traumatic	Hand	None	Flexor digitorum superficialis	Acute
2	33	Male	CAD	Tumour excision	Hand	Multiple prior failed excisions	Extensor digitorum minimi	Acute
3	7	Male	None	Soft tissue infection	Forearm	None	Brachioradialis and flexor carpi radialis	1
4	65	Male	DM, CAD, HTN	Soft tissue infection	Lower leg	None	Tibialis anterior and extensor hallucis	1
5	20	Male	None	Traumatic	Knee	None	Quadriceps and patellar	Acute
6	27	Male	None	Soft tissue infection	Foot	Failed primary closure	Quadratus plantae and flexor digitorum longus	Acute
7	67	Female	DM, HTN	Traumatic	Lower leg	Failed fasciocutaneous flap	Achilles	4
8	35	Female	None	Traumatic	Foot	Failed fasciocutaneous flap	Achilles	2
9	55	Male	Dystrophic calcification	Traumatic	Lower leg	None	Achilles	13
10	70	Male	DM	Soft tissue infection	Foot	Failed fasciocutaneous flap	Achilles	12
Mean ± SD	40 ± 22	- -	-	-	-	-	-	3 ± 5
Median (IQR)	34 (20, 65)	-	-	-	-	-	-	1 (0, 6)

Abbreviations: SD, standard deviation; IQR, interquartile range; CAD, coronary artery disease; DM, diabetes mellitus; HTN, hypertension.

**Table 2:** Healing outcomes.

Participant ID	Reconstructive approach	Number of product applications	NPWT duration (weeks)	Time to tissue coverage (weeks)	STSG take (%)	Time to healing (weeks)	Complications	Length of follow-up (weeks)	Functional assessment
1	Primary closure^a^	1	NA	NA	NA	1	None	107	Full range of motion Supplementary Video S1
2	Primary closure^a^	1	NA	NA	NA	2	None	31	Mild limitation range of motion Supplementary Video S2
3	Secondary intention	1	NA	3	NA	7	None	44	Full range of motion Supplementary Video S3
4	Staged	2	NA	6	NR	16	None	71	Full range of motion Supplementary Video S4
5	Staged	1	NA	2	100	3	None	58	Full range of motion Supplementary Video S5
6	Staged	1	NA	3	100	4	None	24	Full range of motion
7	Fasciocutaneous flap	1	NA	NA	NA	1	None	27	Full range of motion Supplementary Video S6
8	Staged	1	2	2	100	4	None	6	Full range of motion
9	Primary closure	1	NA	NA	NA	1	None	2	Full range of motion
10	Staged	2	3	3	95	5	None	6	Mild limitation range of motion
Mean ± SD	-	1 ± 0	-	3 ± 1	98 ± 2	4 ± 5	-	37 ± 33	-
Median (IQR)	-	1 (1, 1)	-	3 (2, 4)	100 (96, 100)	4 (1, 6)	-	29 (6, 61)	-

Abbreviations: SD, standard deviation; IQR, interquartile range; NPWT, negative pressure wound therapy; STSG, split-thickness skin graft; NA, not applicable; NR, not reported.

^a^Patients received an OFM graft placed as a circumferential tendon-protective layer.

For those participants that were reconstructed using a staged closure or closure via secondary intention, the median time to granulation tissue coverage of the tendon was 3 (IQR: 2, 4) weeks. The STSG take 1 week post-placement was a median of 100 (IQR: 96, 100) %. One participant (#4) received multiple STSGs due to the size of the wound. Across all defects, the median time from the index surgical procedure to full epithelialization was 4 (IQR: 1, 6) weeks. The median follow-up period was 29 (IQR: 6, 61) weeks. Long-term functional outcome was assessed by the attending surgeon who evaluated tissue pliability and active range of motion (Table 2). Where available, long-term follow-up videos demonstrating the range of motion are provided in Supplementary Videos S1–S6.

### Case example 1

**Figure 1:**
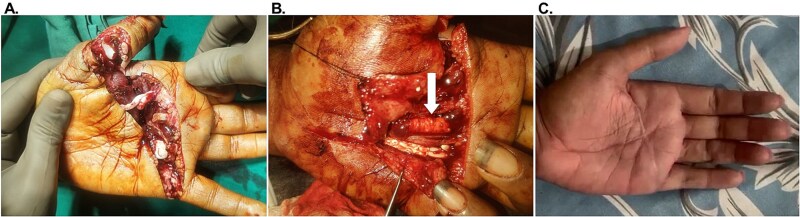
Representative images of Participant #1 (left palm laceration) with exposed flexor digitorum superficialis tendons. (A) Initial presentation. (B) After debridement and placement of the OFM grafts as a circumferential tendon-protective layer to the flexor digitorum superficialis tendons (example indicated with a white arrow). (C) At post-operative week 107.

A 21-year-old male (Table 1, Participant #1) presented with a deep laceration to the left palm following acute hand trauma (Fig 1A). The wound was characterized by exposed flexor tendons in the affected area, with no prior interventions and no significant complicating co-morbidities. OFM grafts (three-layer, 5 × 5 cm) were placed as circumferential tendon-protective layers around exposed tendons of the thumb, index, and middle fingers and did not undergo primary repair (Fig 1B). Tendons of the ring and little finger did not receive any OFM graft, as these required tendon sheath reconstruction. The aim of the circumferential graft placement was to improve tendon gliding and reduce the risk of adhesion formation. The defect was ultimately closed via primary intention. At 1-week follow-up (not shown), the palm was fully healed, with no signs of infection or complications. At 107 weeks, the patient remained well-healed, with sustained functional gains in the treated digits and no recurrence of adhesions or complications (Table 2) (Supplementary Video S1) (Fig 1C).

### Case example 2

**Figure 2:**
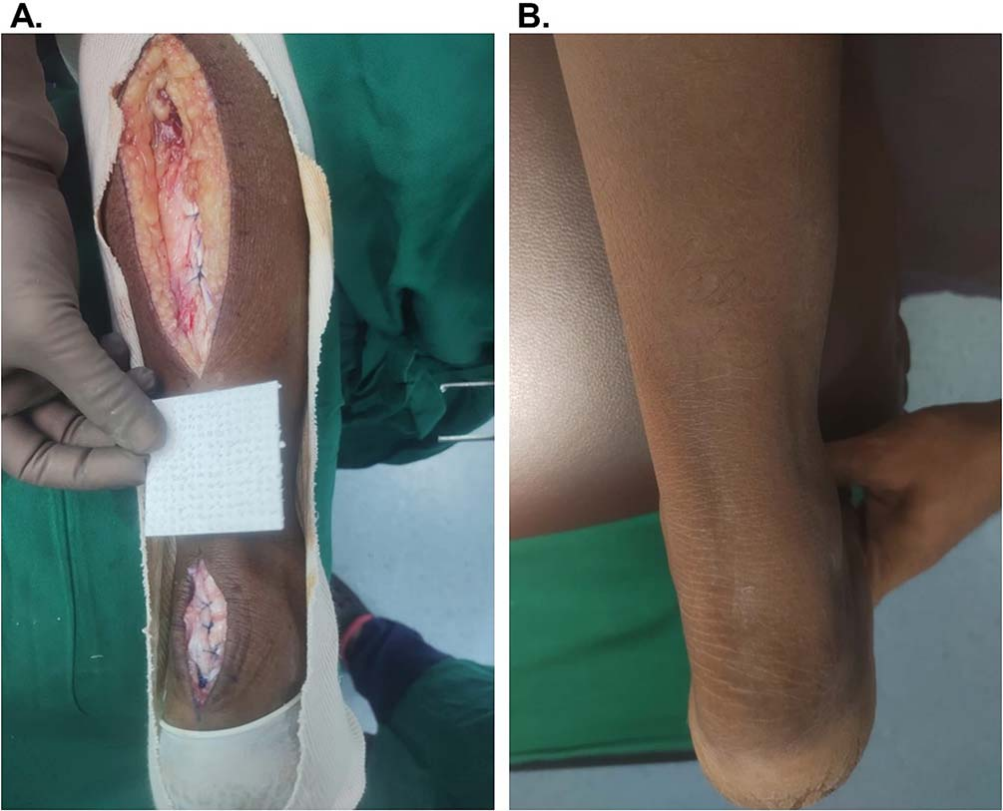
Representative images of Participant #7 (lower extremity, traumatic) with exposed Achilles. (A) After debridement of the defect, prior to OFM placement in the distal incision over the Achilles and flap reconstruction. (B) Week 27, long-term follow-up.

A 67-year-old female (Table 1, Participant #7) with a history of diabetes mellitus and hypertension presented with a subacute lower extremity traumatic defect that had persisted for ~4 weeks. A prior Achilles tendon rupture was complicated by chronic degeneration and calcification, and a reconstruction to close the area with a fasciocutaneous flap had failed. After revision surgery was planned, the affected tendon was exposed and debrided (Fig 2A). An OFM graft (three-layer, 5 × 5 cm) was passed through the proximal incision and placed directly over the Achilles tendon exposed by the distal incision to serve as an adhesion barrier between the tendon and overlying tissue. A turn-down flap was utilized to close the defect. At one week (not shown), the wound demonstrated full healing with no signs of breakdown or infection. At a long-term follow-up of 27 weeks (Fig 2B), the patient demonstrated no signs of tendon tethering or skin adherence (Supplementary Video S6), and no further surgical intervention was required.

## Discussion

In the current case series, OFM grafts were used for tendon coverage to facilitate closure via secondary intention, as an implant under a primary closure or fasciocutaneous flap, and for staged reconstructions. The use of OFM grafts to facilitate granulation tissue coverage over exposed tendons, as part of a staged closure or closure via secondary intention, is well documented [6, 10]. In the current study, OFM supported granulation tissue formation with a median time to coverage of 3 (IQR: 2, 4) weeks, which is consistent with existing published data demonstrating median/average coverage times ranging 23–35 days [8, 16, 18–20]. In this case series, the median STSG take 1 week following application was 100 (IQR: 96, 100) %, indicating that OFM resulted in the development of a supportive, vascularized tissue bed for grafting. STSG take for one patient (Table 2, Participant #4) could not be assessed as he had undergone multiple small STSGs given the size of the defect and his complex medical history. Other bioscaffolds have similarly been explored for tissue coverage over exposed tendons, although with variable times to coverage. For example, for bovine collagen-based products, most clinical studies report vascularized tissue coverage around 4–12 weeks [8, 16, 21].

OFM grafts are additionally indicated for implantation under a primary closure site or fasciocutaneous flap to fill dead space and reduce post-operative complications. This approach has been previously used for the primary closure of various complex chronic wounds, such as pressure injuries [5, 22]. In the current case series, two patients (Table 2) had an OFM graft placed on top of the exposed tendon prior to a fasciocutaneous flap (Participant #7) or primary closure (Participant #9). Both patients healed well, with excellent functional recovery and no complications.

Based on our positive clinical outcomes with OFM grafts in defects involving exposed tendons, we began exploring use into other surgical approaches. For two patients (#1 and #2), OFM grafts were placed circumferentially around the exposed tendons as a protective layer to reduce the risk of post-operative tendon fibrosis, adhesions, and loss of function. Both participants had excellent functional recovery of the affected tendons. Notably, Participant #1 demonstrated significantly improved tendon gliding at the thumb, index, and middle fingers, while developing adhesion and restricted motion in the ring and little fingers, which had not received OFM graft.

Importantly, no post-operative complications were observed in the current cohort, highlighting the safety profile of OFM grafts in contaminated soft tissue defects. Wound dehiscence, hematoma formation, infections, partial graft loss, and diffuse scarring have all been reported when other bioscaffolds were used to cover exposed tendons [23–25]. Regarding cost-effectiveness, the median number of graft applications required across the current cohort was one (range: 1–2), consistent with previous studies. In comparison, and as demonstrated by Lawlor *et al*. [8], some of the commercially available alternatives may require multiple product applications, which increases the overall cost of care.

A key focus of the current case series was to document functional outcomes of patients following reconstruction with OFM grafts. The patients in the current cohort all reported a full, or partial tendon function (Table 2), aligning with a previously published functional assessment [10]. While other bioscaffolds have been successfully used to cover exposed tendons, few studies report quantitative functional outcomes. For example, while the use of acellular dermal matrix or collagen bilayer over exposed tendon has been widely reported, studies that include quantitative functional outcomes are limited [15, 16].

In conclusion, this case series demonstrates the safety and effectiveness of OFM to cover exposed tendons with minimal applications, no post-operative complications, and excellent functional outcomes. Future prospective studies with larger sample sizes and standardized outcome measures are needed to further validate these findings and refine treatment algorithms for complex wounds with exposed tendons.

## Supplementary Material

09_23_25_-_Bosque_-_S_1_rjaf1119

09_23_25_-_Bosque_-_S_2_rjaf1119

09_23_25_-_Bosque_-_S_3_rjaf1119

09_23_25_-_Bosque_-_S_4_rjaf1119

09_23_25_-_Bosque_-_S_5_rjaf1119

09_23_25_-_Bosque_-_S_6_rjaf1119

## References

[1] Deng Z, Long Z-S, Chen G. Mini-review: tendon-exposed wound treatments. Journal of Investigative Surgery 2023;36:2266758. 10.1080/08941939.2023.2266758 37813390

[2] Lin CT, Chen SG, Chen TM, et al. Bipedicled flap for the reconstruction of soft tissue defects of the Achilles tendon. Ann Plast Surg 2015;74:484–7. 10.1097/SAP.0b013e3182a1e508 25760483

[3] Shores JT, Hiersche M, Gabriel A, et al. Tendon coverage using an artificial skin substitute. J Plast Reconstr Aesthet Surg 2012;65:1544–50. 10.1016/j.bjps.2012.05.021 22721977

[4] Chia J, Lim A, Peng Y-P. Use of an arterialized venous flap for resurfacing a circumferential soft tissue defect of a digit. Microsurgery 2001;21:374–8. 10.1002/micr.21805 11757064

[5] Desvigne MN, Bauer K, Holifield K, et al. Case report: surgical closure of chronic soft tissue defects using extracellular matrix graft augmented tissue flaps. Front Surg 2020;7:559450. 10.3389/fsurg.2020.559450 33575271 PMC7871006

[6] Chaffin AE, Dowling SG, Kosyk MS, et al. Surgical reconstruction of pilonidal sinus disease with concomitant extracellular matrix graft placement: a case series. J Wound Care 2021;30:S28–34. 10.12968/jowc.2021.30.Sup7.S28 34256587

[7] Sadtler K, Wolf MT, Ganguly S, et al. Divergent immune responses to synthetic and biological scaffolds. Biomaterials 2019;192:405–15. 10.1016/j.biomaterials.2018.11.002 30500722

[8] Lawlor JC, Bosque BA, Frampton C, et al. Limb salvage via surgical soft-tissue reconstruction with ovine forestomach matrix grafts: a prospective study. PRS Glob Open 2024;12:e6406. 10.1097/GOX.0000000000006406 PMC11661765 39712384

[9] Bosque BA, Dowling SG, May BCH, et al. Ovine forestomach matrix in the surgical management of complex lower-extremity soft-tissue defects: a retrospective multi-center case series. J Am Podiatr Med Assoc 2023;113:22–081. 10.7547/22-081 37463196

[10] Duplechain AB, Bosque BA, Fligor CW, et al. Soft tissue reconstruction with ovine forestomach matrix after wide excision of plantar fibromatosis. ePlasty 2023;2023:e20. PMC10176481 37187868

[11] Bohn GA, Chaffin AE. Extracellular matrix graft for reconstruction over exposed structures: a pilot case series. J Wound Care 2020;29:742–9. 10.12968/jowc.2020.29.12.742 33320746

[12] Lun S, Irvine SM, Johnson KD, et al. A functional extracellular matrix biomaterial derived from ovine forestomach. Biomaterials 2010;31:4517–29. 10.1016/j.biomaterials.2010.02.025 20226520

[13] Irvine SM, Cayzer J, Todd EM, et al. Quantification of in vitro and in vivo angiogenesis stimulated by ovine forestomach matrix biomaterial. Biomaterials 2011;32:6351–61. 10.1016/j.biomaterials.2011.05.040 21665268

[14] Smith MJ, Dempsey SG, Veale RWF, et al. Further structural characterization of ovine forestomach matrix and multi-layered extracellular matrix composites for soft tissue repair. J Biomater Appl 2021;36:996–1010. 10.1177/08853282211045770 34747247 PMC8721687

[15] Dardano AN, Efimenko I, Florio T, et al. Rapid revascularization following application of ovine forestomach matrix graft in complex facial and scalp trauma. Trauma Cases Rev 2024;10:105. 10.23937/2469-5777/1510105

[16] Cormican MT, Creel NJ, Bosque BA, et al. Ovine forestomach matrix in the surgical management of complex volumetric soft tissue defects: a retrospective pilot case series. ePlasty 2023;23:e66. 38045101 PMC10690777

[17] Nadafpour N, Montazeri M, Moradi M, et al. Bacterial colonization on different suture materials used in oral implantology: a randomized clinical trial. Front Dent 2021;18:25. 10.18502/fid.v18i25.6935 35965706 PMC9355897

[18] Mundra LS, Tucker NJ, Parry JA. Urinary bladder matrix grafting versus flap coverage for acute or infected wound defects in patients with orthopaedic trauma. J Orthop Trauma 2022;36:e374–9. 10.1097/BOT.0000000000002406 35580325

[19] Parry JA, Shannon SF, Strage KE, et al. Urinary bladder matrix grafting: a simple and effective alternative to flap coverage for wounds in high-risk orthopaedic trauma patients. J Orthop Trauma 2021;36:e152–7. 10.1097/BOT.0000000000002245 34417765

[20] Shakir S, Messa CA, Broach RB, et al. Indications and limitations of bilayer wound matrix-based lower extremity reconstruction: a multidisciplinary case-control study of 191 wounds. Plast Reconstr Surg 2020;145:813–22. 10.1097/PRS.0000000000006609 32097330 PMC7043722

[21] Bosque BA, Dowling SG, May BCH, et al. Ovine forestomach matrix in the surgical management of complex lower-extremity soft-tissue defects. J Am Podiatr Med Assoc 2023;113:22–081. 10.7547/22-081 37463196

[22] Nasseri Y, Oka K, la K, et al. Ovine forestomach matrix graft reduces surgical dehiscence in fasciocutaneous flap-based closure of pilonidal disease: a comparative study. Cureus 2025;17:e96775. 10.7759/cureus.96775 41245926 PMC12618154

[23] Geiger SE, Deigni OA, Watson JT, et al. Management of open distal lower extremity wounds with exposed tendons using porcine urinary bladder matrix. Wounds 2016;28:306–16. 27701126

[24] Marchesi A, Parodi PC, Brioschi M, et al. Soft-tissue defects of the Achilles tendon region: management and reconstructive ladder. Review of the literature. Injury 2016;47:S147–53. 10.1016/j.injury.2016.07.053 27492062

[25] Tao M, Liang F, He J, et al. Decellularized tendon matrix membranes prevent post-surgical tendon adhesion and promote functional repair. Acta Biomater 2021;134:160–76. 10.1016/j.actbio.2021.07.038 34303866

